# Ni_3_Te_2_O_2_(PO_4_)_2_(OH)_4_, an open-framework structure isotypic with Co_3_Te_2_O_2_(PO_4_)_2_(OH)_4_

**DOI:** 10.1107/S2056989020004466

**Published:** 2020-04-03

**Authors:** Felix Eder, Matthias Weil

**Affiliations:** a Institute for Chemical Technologies and Analytics, Division of Structural Chemistry, TU Wien, Getreidemarkt 9/164-SC, A-1060 Vienna, Austria

**Keywords:** crystal structure, oxidotellurate(IV), phosphate, framework structure, isotypism, structure comparison

## Abstract

Single crystals of Ni_3_(TeO(OH)_2_)_2_(PO_4_)_2_, trinickel(II) bis[(oxidodihydoxidotellurate(IV)] bis(phosphate),were obtained by hydro­thermal synthesis at 483 K, starting from NiCO_3_·2Ni(OH)_2_, TeO_2_ and H_3_PO_4_ in a molar ratio of 1:2:2. The crystal structure of Ni_3_Te_2_O_2_(PO_4_)_2_(OH)_4_ is isotypic with that of Co_3_Te_2_O_2_(PO_4_)_2_(OH)_4_ [Zimmermann *et al.* (2011 ▸). *J. Solid State Chem*. **184**, 3080–3084]. The asymmetric unit comprises two Ni (site symmetries , 2/*m*) one Te (*m*), one P (*m*), five O (three *m*, two 1) and one H (1) sites. The tellurium(IV) atom shows a coordination number of five, with the corresponding [TeO_3_(OH)_2_] polyhedron having a distorted square-pyramidal shape. The two Ni^II^ atoms are both octa­hedrally coordinated but form different structural elements: one constitutes chains made up from edge-sharing [NiO_6_] octa­hedra extending parallel to [010], and the other isolated [NiO_2_(OH)_4_] octa­hedra. The two kinds of nickel/oxygen octa­hedra are connected by the [TeO_3_(OH)_2_] pyramids and the [PO_4_] tetra­hedra through edge- and corner-sharing into a three-dimensional framework structure with channels extending parallel to [010]. Hydrogen bonds of medium strength between the hy­droxy groups and one of the phosphate O atoms consolidate the packing. A qu­anti­tative structure comparison between Ni_3_Te_2_O_2_(PO_4_)_2_(OH)_4_ and Co_3_Te_2_O_2_(PO_4_)_2_(OH)_4_ is made.

## Abstract 2

The crystal structure of Ni_3_Te_2_O_2_(PO_4_)_2_(OH)_4_ comprises a comparatively rare penta-coordinated Te^IV^ atom, resulting in a [TeO_3_(OH)_2_] square-pyramidal coordination polyhedron.

## Chemical context   

The crystal chemistry of Te^IV^-containing compounds is very diverse and strongly influenced by the stereochemically active 5*s* ^2^ lone pair. The space requirement of the latter frequently results in one-sided and low-symmetric coordination spheres around Te^IV^, as surveyed recently for the vast family of oxidotellurates(IV) (Christy *et al.*, 2016 ▸). The polarities and shapes of corresponding oxidotellurate(IV) anions can be utilized in the search for new compounds with non-centrosymmetric structures. The absence of an inversion centre is a precondition for a substance to have ferro-, pyro- or piezoelectric properties or to have non-linear optical properties (Ok *et al.*, 2006 ▸). Combining oxidotellurates(IV) with additional (transition) metal cations often leads to open-framework structures because of the space required for the 5*s* ^2^ lone pair. This way, either channels can be integrated within three-dimensional frameworks, or layers, chains or clusters of building blocks can be formed (Stöger & Weil, 2013 ▸). Incorporating additional anions into transition-metal oxidotellurates(IV) increases the possibilities for structural diversification. Following this strategy, several mixed-anion oxotellurates(IV) have been characterized over the last decade, including sulfates [*e.g.* Cd_4_(SO_4_)(TeO_3_)_3_; Weil & Shirkhanlou, 2017 ▸], selenates [*e.g*. Hg_3_(SeO_4_)(TeO_3_); Weil & Shirkhanlou, 2015 ▸], nitrates [*e.g.* Ca_6_Te_5_O_15_(NO_3_)_2_; Stöger & Weil, 2013 ▸] or phosphates [*e.g.* Co_3_Te_2_O_2_(PO_4_)_2_(OH)_4_; Zimmermann *et al.*, 2011 ▸].

In this communication we describe the synthesis and crystal structure analysis of Ni_3_Te_2_O_2_(PO_4_)_2_(OH)_4_, which is isotypic with its cobalt(II) analogue. The two structures are qu­anti­tatively compared.

## Structural commentary   

Of the ten atoms in the asymmetric unit (2 Ni, 1 Te, 1 P, 5 O, 1 H), seven are situated on special positions. Ni1 is located on an inversion centre (Wyckoff position 4 *f*), Ni2 on a site with symmetry 2/*m* (2 *b*), Te1 and P1 both possess site symmetry *m* (4 *i*), and three of the oxygen sites (O1, O3, O4) are likewise located on a mirror plane (4 *i*) while the other two oxygen atoms and the hydrogen atom (O2, O5, H1) are located on general positions (8 *j*).

Both nickel atoms are coordinated octa­hedrally but otherwise show a different environment. Ni1 is surrounded by six oxygen atoms (O1, O2, O3 and their symmetry-related counterparts) and forms chains of edge-sharing [Ni1O_6_] octa­hedra extending parallel to [010]. The ^1^_∞_[Ni1O_4/2_O_2/1_] chains are not entirely straight; the octa­hedra are tilted against each other with every second unit being oriented in the same direction. The three pairs of Ni1—O bond lengths are rather similar (Table 1 ▸), with an average length of 2.058 Å. The distance between neighbouring Ni1 atoms in a chains amounts to 2.9717 (11) Å. Ni2 is coordinated by two O atoms (O4 and its symmetry-related counterpart) in axial positions and by four hydroxide groups (O5 and its three symmetry-related counterparts) in the equatorial positions. The latter have a slightly longer bond than the former (Δ = 0.032 Å), with an average bond length of 2.084 Å for the six O atoms. The [Ni2O_2_(OH)_4_] octa­hedra are isolated from each other and are also not linked to the ^1^_∞_[Ni1O_4/2_O_2/1_] chains.

**Figure 1:**
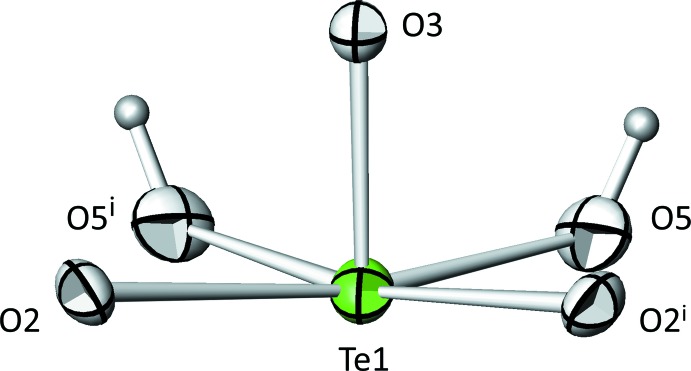
The square-pyramidal [TeO_3_(OH)_2_] polyhedron in the title compound. Displacement ellipsoids are drawn at the 90% probability level. [Symmetry code: (i) *x*, −*y* + 1, *z*.]

**Figure 2:**
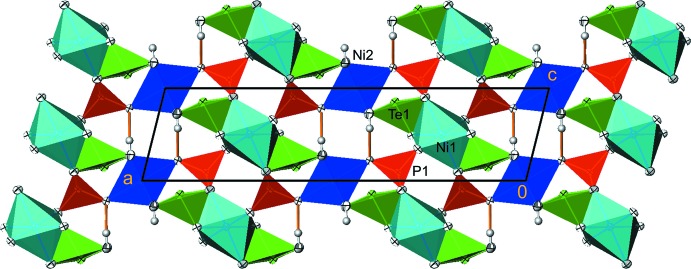
The crystal structure of Ni_3_Te_2_O_2_(PO_4_)_2_(OH)_4_ in a projection along [010]. Displacement ellipsoids are drawn at the 90% probability level; hydrogen bonds are shown as orange lines.

Te1 is coordinated by five oxygen atoms, two of them being hydroxide groups. The surrounding atoms form a distorted square pyramid (Fig. 1 ▸), a coordination polyhedron that is comparatively rare in the crystal chemistry of oxidotellurates(IV), with a trigonal pyramid (TeO_3_^2–^) as the most commonly observed type of anion (Christy *et al.*, 2016 ▸). The Te1 atom is displaced from the basal plane of the pyramid by 0.1966 (2) Å. The two symmetry-related O2 atoms defining one side of the basal plane exhibit a significantly larger distance [2.3094 (18) Å] from Te than the two hy­droxy groups [2.0002 (18) Å] on the other side. The oxygen atom closest [O3, 1.866 (2) Å] to Te1 lies at the apex of the pyramid. The calculated bond-valence sum (BVS; Brown, 2002 ▸) of Te1 is 4.05 valence units (v.u.) based on the parameters of Brese & O’Keeffe (1991 ▸). Using the revised parameters of Mills & Christy (2013 ▸), a BVS of 3.93 v.u. was calculated. The [TeO_3_(OH)_2_] units are not connected to each other but share two edges with [Ni1O_6_] octa­hedra and two corners, being the hydroxide groups, with [Ni2O_2_(OH)_4_] octa­hedra, as well as a corner of the phosphate tetra­hedra. In this way, a three-dimensional framework structure is obtained with channels running parallel to [010]. The free-electron pairs point into the smaller type of channels whereas the hydrogen atoms of the hy­droxy group protrude into the larger type of channels. This results in hydrogen bonds of medium strength, with the OH groups linking to opposite O atoms (Fig. 2 ▸; Table 2 ▸).

As a result of the similar ionic radii (Shannon, 1976 ▸) of six-coordinated Ni^2+^ (0.69 Å) and Co^2+^ (0.75 Å, assuming a high-spin *d* ^7^ state), the comparable bond lengths in the two isotypic structures differ only marginally (Table 1 ▸). The two structures were also qu­anti­tatively compared using the program *compstru* (de la Flor *et al.*, 2016 ▸). The absolute distances between paired atoms are 0 Å for Ni1/Co1, 0 Å for Ni2/Co2, 0.0213 Å for P1, 0.0289 Å for O1, 0.0289 Å for O2, 0.0342 Å for O3, 0.0192 Å for O4 and 0.0271 Å for O5. The degree of lattice distortion is 0.0072, the arithmetic mean of the distance between paired atoms is 0.0227 Å, and the measure of similarity is 0.011.

## Synthesis and crystallization   

Crystals of Ni_3_Te_2_O_2_(PO_4_)_2_(OH)_4_ were obtained under hydro­thermal conditions. The starting materials, 0.1796 g (0.591 mmol) NiCO_3_·2Ni(OH)_2_, 0.1870 g TeO_2_ (1.172 mmol) and 0.16 g 85% H_3_PO_4_ (1.4 mmol), were weighed into a small Teflon vessel with a volume of *ca* 3 ml. The reactants were mixed, and the vessel filled to about two thirds with deionized water. The reaction vessel was heated inside a steel autoclave at 483 K for 7 d; the autoclave was removed from the oven and allowed to cool to room temperature over about four hours. A bright-green solid besides small amounts of a pale-yellow powder was obtained as the reaction product. X-ray powder diffraction of the bulk revealed Ni_3_Te_2_O_2_(PO_4_)_2_(OH)_4_ as the main product and TeO_2_ (corresponding to the pale-yellow powder) as a side product. A light-green block-shaped single crystal of Ni_3_Te_2_O_2_(PO_4_)_2_(OH)_4_ was selected for the diffraction experiment.

## Refinement   

Crystal data, data collection and structure refinement details are summarized in Table 3 ▸. Atom labels and starting coordinates for refinement were adopted from the isotypic Co_3_Te_2_O_2_(PO_4_)_2_(OH)_4_ structure (Zimmermann *et al.*, 2011 ▸). The hydrogen atom of the hy­droxy group was located in a difference-Fourier map and was refined freely. The remaining maximum electron density of 3.6 e^−^ Å^−3^ is located 0.71 Å from P1. Modelling the corresponding site as a minor disorder component lead to unrealistic P—O distances and physically non-reasonable displacement parameters. We therefore did not consider this site in the final model.

## Supplementary Material

Crystal structure: contains datablock(s) I. DOI: 10.1107/S2056989020004466/hb7902sup1.cif

Structure factors: contains datablock(s) I. DOI: 10.1107/S2056989020004466/hb7902Isup2.hkl

CCDC reference: 1993934

Additional supporting information:  crystallographic information; 3D view; checkCIF report

## Figures and Tables

**Table 1:** Comparison of bond lengths (Å) in the isotypic *M*_3_Te_2_O_2_(PO_4_)_2_(OH)_4_ compounds (*M* = Ni, Co*^*a*^*)

	*M* = Ni	*M* = Co
Te1—O3	1.866 (2)	1.861 (4)
Te1—O5	2.0002 (18)	1.994 (3)
Te1—O2	2.3093 (18)	2.331 (3)
*M*1—O1	2.0343 (16)	2.051 (3)
*M*1—O2	2.0457 (18)	2.079 (3)
*M*1—O3	2.0940 (18)	2.143 (3)
*M*2—O4	2.061 (2)	2.076 (4)
*M*2—O5	2.0928 (19)	2.147 (3)
P1—O4	1.534 (3)	1.530 (4)
P1—O1	1.538 (2)	1.541 (5)
P1—O2	1.5449 (18)	1.550 (3)

(*a*) Unit-cell parameters: *a* = 19.4317 (10), *b* = 6.0249 (3), *c* = 4.7788 (2) Å, *β* = 103.139 (5)°, *V* = 544.83 (5) Å^3^ (Zimmermann *et al.*, 2011 ▸).

**Table 2:** Hydrogen-bond geometry (Å, °)

*D*—H⋯*A*	*D*—H	H⋯*A*	*D*⋯*A*	*D*—H⋯*A*
O5—H1⋯O4^i^	0.80 (5)	2.03 (5)	2.812 (3)	164 (6)

Symmetry code: (i) 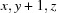.

**Table 3:** Experimental details

Crystal data	
Chemical formula	Ni_3_Te_2_O_2_(PO_4_)_2_(OH)_4_
*M*_r_	721.30
Crystal system, space group	Monoclinic, *C*2/*m*
Temperature (K)	300
*a*, *b*, *c* (Å)	19.241 (7), 5.943 (2), 4.7808 (18)
β (°)	104.094 (8)
*V* (Å^3^)	530.3 (3)
*Z*	2
Radiation type	Mo *K*α
μ (mm^−1^)	11.05
Crystal size (mm)	0.08 × 0.06 × 0.03

Data collection	
Diffractometer	Bruker APEXII CCD
Absorption correction	Multi-scan (*SADABS*; Bruker, 2016 ▸)
*T*_min_, *T*_max_	0.600, 0.747
No. of measured, independent and observed [*I* > 2σ(*I*)] reflections	12917, 1292, 1212
*R*_int_	0.054
(sin θ/λ)_max_ (Å^−1^)	0.820

Refinement	
*R*[*F* ^2^ > 2σ(*F* ^2^)], *wR*(*F* ^2^), *S*	0.022, 0.058, 1.11
No. of reflections	1292
No. of parameters	63
H-atom treatment	All H-atom parameters refined
Δρ_max_, Δρ_min_ (e Å^−3^)	3.84, −1.39

Computer programs: *APEX3* and *SAINT* (Bruker, 2016 ▸), *SHELXL* (Sheldrick, 2015 ▸), *ATOMS* (Dowty, 2006 ▸) and *publCIF* (Westrip, 2010 ▸).

## supplementary crystallographic information

### Crystal data

**Table:**

Ni_3_Te_2_O_2_(PO_4_)_2_(OH)_4_	*F*(000) = 668
*M**_r_* = 721.30	*D*_x_ = 4.518 Mg m^−^^3^
Monoclinic, *C*2/*m*	Mo *K*α radiation, λ = 0.71073 Å
*a* = 19.241 (7) Å	Cell parameters from 8089 reflections
*b* = 5.943 (2) Å	θ = 3.6–35.3°
*c* = 4.7808 (18) Å	µ = 11.05 mm^−^^1^
β = 104.094 (8)°	*T* = 300 K
*V* = 530.3 (3) Å^3^	Block, light green
*Z* = 2	0.08 × 0.06 × 0.03 mm

### Data collection

**Table:**

Bruker APEXII CCD diffractometer	1212 reflections with *I* > 2σ(*I*)
ω– and φ–scan	*R*_int_ = 0.054
Absorption correction: multi-scan (*SADABS*; Bruker, 2016)	θ_max_ = 35.7°, θ_min_ = 3.6°
*T*_min_ = 0.600, *T*_max_ = 0.747	*h* = −31→31
12917 measured reflections	*k* = −9→9
1292 independent reflections	*l* = −7→7

### Refinement

**Table:**

Refinement on *F*^2^	Primary atom site location: isomorphous structure methods
Least-squares matrix: full	Hydrogen site location: difference Fourier map
*R*[*F*^2^ > 2σ(*F*^2^)] = 0.022	All H-atom parameters refined
*wR*(*F*^2^) = 0.058	*w* = 1/[σ^2^(*F*_o_^2^) + (0.0264*P*)^2^ + 2.8929*P*] where *P* = (*F*_o_^2^ + 2*F*_c_^2^)/3
*S* = 1.11	(Δ/σ)_max_ = 0.001
1292 reflections	Δρ_max_ = 3.84 e Å^−^^3^
63 parameters	Δρ_min_ = −1.39 e Å^−^^3^
0 restraints	

### Special details

**Table:**

Geometry. All esds (except the esd in the dihedral angle between two l.s. planes) are estimated using the full covariance matrix. The cell esds are taken into account individually in the estimation of esds in distances, angles and torsion angles; correlations between esds in cell parameters are only used when they are defined by crystal symmetry. An approximate (isotropic) treatment of cell esds is used for estimating esds involving l.s. planes.

### Fractional atomic coordinates and isotropic or equivalent isotropic displacement parameters (Å^2^)

**Table:**

	*x*	*y*	*z*	*U*_iso_*/*U*_eq_	
Te1	0.39329 (2)	0.500000	0.84942 (4)	0.00797 (6)	
Ni1	0.250000	0.250000	0.500000	0.00608 (8)	
Ni2	0.500000	1.000000	1.000000	0.00680 (11)	
P1	0.33875 (4)	0.000000	0.09068 (16)	0.00516 (13)	
O1	0.30175 (13)	0.000000	0.3417 (5)	0.0075 (4)	
O2	0.31714 (9)	0.2109 (3)	0.9006 (4)	0.0085 (3)	
O3	0.32631 (13)	0.500000	0.4940 (5)	0.0075 (4)	
O4	0.42002 (13)	0.000000	0.2189 (5)	0.0093 (4)	
O5	0.45243 (10)	0.7393 (3)	0.7251 (4)	0.0110 (3)	
H1	0.441 (3)	0.790 (10)	0.565 (11)	0.043 (14)*	

### Atomic displacement parameters (Å^2^)

**Table:**

	*U*^11^	*U*^22^	*U*^33^	*U*^12^	*U*^13^	*U*^23^
Te1	0.00681 (10)	0.00738 (9)	0.00868 (10)	0.000	−0.00009 (7)	0.000
Ni1	0.00630 (17)	0.00493 (16)	0.00686 (16)	0.00020 (12)	0.00132 (13)	−0.00027 (12)
Ni2	0.0063 (2)	0.0062 (2)	0.0081 (2)	0.000	0.00213 (19)	0.000
P1	0.0061 (3)	0.0051 (3)	0.0049 (3)	0.000	0.0026 (2)	0.000
O1	0.0095 (10)	0.0062 (9)	0.0084 (9)	0.000	0.0051 (8)	0.000
O2	0.0094 (7)	0.0071 (6)	0.0084 (6)	−0.0011 (5)	0.0009 (5)	0.0023 (5)
O3	0.0085 (10)	0.0060 (9)	0.0074 (9)	0.000	0.0008 (8)	0.000
O4	0.0062 (9)	0.0137 (10)	0.0077 (9)	0.000	0.0013 (7)	0.000
O5	0.0098 (7)	0.0118 (7)	0.0109 (7)	−0.0046 (6)	0.0017 (6)	−0.0004 (6)

### Geometric parameters (Å, º)

**Table:**

Te1—O3	1.866 (2)	Ni1—Ni1^iv^	2.9717 (11)
Te1—O5	2.0002 (18)	Ni2—O4^v^	2.061 (2)
Te1—O5^i^	2.0002 (18)	Ni2—O4^vi^	2.061 (2)
Te1—O2^i^	2.3093 (18)	Ni2—O5^vii^	2.0928 (19)
Te1—O2	2.3094 (18)	Ni2—O5^viii^	2.0928 (19)
Ni1—O1	2.0343 (16)	Ni2—O5^ix^	2.0928 (19)
Ni1—O1^ii^	2.0343 (16)	Ni2—O5	2.0928 (19)
Ni1—O2^ii^	2.0457 (18)	P1—O4	1.534 (3)
Ni1—O2	2.0457 (18)	P1—O1	1.538 (2)
Ni1—O3^ii^	2.0940 (18)	P1—O2^x^	1.5449 (18)
Ni1—O3	2.0940 (18)	P1—O2^xi^	1.5449 (18)
Ni1—Ni1^iii^	2.9717 (11)		
			
O3—Te1—O5	92.64 (8)	O4^vi^—Ni2—O5^viii^	93.01 (7)
O3—Te1—O5^i^	92.64 (8)	O5^vii^—Ni2—O5^viii^	84.49 (11)
O5—Te1—O5^i^	90.65 (11)	O4^v^—Ni2—O5^ix^	93.01 (7)
O3—Te1—O2^i^	77.35 (7)	O4^vi^—Ni2—O5^ix^	86.99 (7)
O5—Te1—O2^i^	85.64 (8)	O5^vii^—Ni2—O5^ix^	95.51 (11)
O5^i^—Te1—O2^i^	169.13 (7)	O5^viii^—Ni2—O5^ix^	180.0
O3—Te1—O2	77.35 (7)	O4^v^—Ni2—O5	86.99 (7)
O5—Te1—O2	169.13 (7)	O4^vi^—Ni2—O5	93.01 (7)
O5^i^—Te1—O2	85.64 (8)	O5^vii^—Ni2—O5	180.0
O2^i^—Te1—O2	96.14 (9)	O5^viii^—Ni2—O5	95.51 (11)
O1—Ni1—O1^ii^	180.0	O5^ix^—Ni2—O5	84.49 (11)
O1—Ni1—O2^ii^	89.40 (9)	O4—P1—O1	107.98 (14)
O1^ii^—Ni1—O2^ii^	90.60 (9)	O4—P1—O2^x^	109.71 (9)
O1—Ni1—O2	90.60 (9)	O1—P1—O2^x^	110.48 (9)
O1^ii^—Ni1—O2	89.40 (9)	O4—P1—O2^xi^	109.71 (9)
O2^ii^—Ni1—O2	180.0	O1—P1—O2^xi^	110.48 (9)
O1—Ni1—O3^ii^	83.98 (8)	O2^x^—P1—O2^xi^	108.48 (14)
O1^ii^—Ni1—O3^ii^	96.02 (8)	P1—O1—Ni1^iv^	130.69 (6)
O2^ii^—Ni1—O3^ii^	78.95 (8)	P1—O1—Ni1	130.69 (6)
O2—Ni1—O3^ii^	101.05 (8)	Ni1^iv^—O1—Ni1	93.84 (10)
O1—Ni1—O3	96.02 (8)	P1^xii^—O2—Ni1	131.38 (11)
O1^ii^—Ni1—O3	83.98 (8)	P1^xii^—O2—Te1	125.39 (10)
O2^ii^—Ni1—O3	101.05 (8)	Ni1—O2—Te1	95.09 (6)
O2—Ni1—O3	78.95 (8)	Te1—O3—Ni1	108.58 (9)
O3^ii^—Ni1—O3	180.0	Te1—O3—Ni1^iii^	108.58 (9)
O4^v^—Ni2—O4^vi^	180.00 (7)	Ni1—O3—Ni1^iii^	90.40 (10)
O4^v^—Ni2—O5^vii^	93.01 (7)	P1—O4—Ni2^xiii^	127.69 (15)
O4^vi^—Ni2—O5^vii^	86.99 (7)	Te1—O5—Ni2	122.22 (9)
O4^v^—Ni2—O5^viii^	86.99 (7)		

Symmetry codes: (i) *x*, −*y*+1, *z*; (ii) −*x*+1/2, −*y*+1/2, −*z*+1; (iii) −*x*+1/2, *y*+1/2, −*z*+1; (iv) −*x*+1/2, *y*−1/2, −*z*+1; (v) −*x*+1, −*y*+1, −*z*+1; (vi) *x*, *y*+1, *z*+1; (vii) −*x*+1, −*y*+2, −*z*+2; (viii) *x*, −*y*+2, *z*; (ix) −*x*+1, *y*, −*z*+2; (x) *x*, −*y*, *z*−1; (xi) *x*, *y*, *z*−1; (xii) *x*, *y*, *z*+1; (xiii) *x*, *y*−1, *z*−1.

### Hydrogen-bond geometry (Å, º)

**Table:**

*D*—H···*A*	*D*—H	H···*A*	*D*···*A*	*D*—H···*A*
O5—H1···O4^xiv^	0.80 (5)	2.03 (5)	2.812 (3)	164 (6)

Symmetry code: (xiv) *x*, *y*+1, *z*.
